# Shock Index in the early assessment of febrile children at the emergency department: a prospective multicentre study

**DOI:** 10.1136/archdischild-2020-320992

**Published:** 2021-06-22

**Authors:** Nienke N Hagedoorn, Joany M Zachariasse, Dorine Borensztajn, Elise Adriaansens, Ulrich von Both, Enitan D Carrol, Irini Eleftheriou, Marieke Emonts, Michiel van der Flier, Ronald de Groot, Jethro Adam Herberg, Benno Kohlmaier, Emma Lim, Ian Maconochie, Federico Martinón-Torres, Ruud Gerard Nijman, Marko Pokorn, Irene Rivero-Calle, Maria Tsolia, Dace Zavadska, Werner Zenz, Michael Levin, Clementien Vermont, Henriette A Moll, the PERFORM consortium

**Affiliations:** 1 Department of Pediatrics, Erasmus MC Sophia, Rotterdam, The Netherlands; 2 General Paediatrics, Erasmus MC Sophia Children's Hospital, Rotterdam, The Netherlands; 3 Department of Pediatrics, Erasmus MC Sophia Children's Hospital, Rotterdam, The Netherlands; 4 Division of Paediatric Infectious Diseases, Dr von Haunersches Children's Hospital, Children's Clinic and Children's Polyclinic of the Ludwig Maximilian University of Munich, Munchen, Germany; 5 Partner Site Munich, German Centre for Infection Research, Braunschweig, Germany; 6 Institute of Infection, Veterinary and Ecological Sciences, Global Health Liverpool, University of Liverpool, Liverpool, UK; 7 Paediatric Infectious Diseases and Immunology, Alder Hey Children's NHS Foundation Trust, Liverpool, UK; 8 Second Department of Paediatrics, P and A Kyriakou Children’s Hospital, National and Kapodistrian University of Athens, Athens, Greece; 9 Paediatric Immunology, Infectious Diseases and Allergy, Great North Children's Hospital, Newcastle Upon Tyne Hospitals NHS Foundation Trust, Newcastle Upon Tyne, UK; 10 Translational and Clinical Research Institute, Newcastle University, Newcastle upon Tyne, UK; 11 Pediatric Infectious Diseases and Immunology, Amalia Children's Hospital, Radboud University Medical Center, Nijmegen, The Netherlands; 12 Section of Paediatric Infectious Diseases, Laboratory of Medical Immunology, Radboud University, Radboud Institute for Molecular Life Sciences, Nijmegen, The Netherlands; 13 Division of Paediatric Infectious Diseases, Imperial College London, London, UK; 14 Department of General Paediatrics, Medical University of Graz, Graz, Austria; 15 Paediatric Emergency Medicine, Imperial College Healthcare NHS Trust, London, UK; 16 Genetics, Vaccines, Infections and Paediatrics Research Group (GENVIP), University Hospital of Santiago de Compostela, Santiago de Compostela, Spain; 17 Department of Infectious Diseases, Ljubljana University Clinical Center, Ljubljana, Slovenia; 18 Department of Pediatrics, Rigas Stradinas University, Riga, Latvia; 19 Department of Paediatric Infectious Diseases and Immunology, Erasmus MC Sophia Children's Hospital, Rotterdam, The Netherlands

**Keywords:** epidemiology, physiology

## Abstract

**Objective:**

(1) To derive reference values for the Shock Index (heart rate/systolic blood pressure) based on a large emergency department (ED) population of febrile children and (2) to determine the diagnostic value of the Shock Index for serious illness in febrile children.

**Design/setting:**

Observational study in 11 European EDs (2017–2018).

**Patients:**

Febrile children with measured blood pressure.

**Main outcome measures:**

Serious bacterial infection (SBI), invasive bacterial infection (IBI), immediate life-saving interventions (ILSIs) and intensive care unit (ICU) admission. The association between high Shock Index (>95th centile) and each outcome was determined by logistic regression adjusted for age, sex, referral, comorbidity and temperature. Additionally, we calculated sensitivity, specificity and negative/positive likelihood ratios (LRs).

**Results:**

Of 5622 children, 461 (8.2%) had SBI, 46 (0.8%) had IBI, 203 (3.6%) were treated with ILSI and 69 (1.2%) were ICU admitted. High Shock Index was associated with SBI (adjusted OR (aOR) 1.6 (95% CI 1.3 to 1.9)), ILSI (aOR 2.5 (95% CI 2.0 to 2.9)), ICU admission (aOR 2.2 (95% CI 1.4 to 2.9)) but not with IBI (aOR: 1.5 (95% CI 0.6 to 2.4)). For the different outcomes, sensitivity for high Shock Index ranged from 0.10 to 0.15, specificity ranged from 0.95 to 0.95, negative LRs ranged from 0.90 to 0.95 and positive LRs ranged from 1.8 to 2.8.

**Conclusions:**

High Shock Index is associated with serious illness in febrile children. However, its rule-out value is insufficient which suggests that the Shock Index is not valuable as a screening tool for all febrile children at the ED.

## Abstract (precis)

EU multicentre ED study of presenting vital signs in >5000 febrile children with an association between "shock index" and serious illness, but low sensitivity limits utility in screening.

**What is already known on this topic?**

- Shock Index (heart rate/systolic blood pressure) is a proposed non-invasive measure for haemodynamic assessment.
- In children, high Shock Index is associated with major trauma and hospitalisation following emergency department (ED) visit.
- Shock Index reference values and the value of the Shock Index to identify serious illness for febrile children attending the ED are unknown.

**What this study adds?**

- In this cohort of febrile children at the ED, we provide reference values for the Shock Index.
- High Shock Index is associated with serious illness in febrile children, but its low sensitivity makes it not valuable as a screening tool.
- Our study suggests that the Shock Index is not valuable as a routine screening tool in the early assessment of febrile children at the ED.

## Background

Early recognition of serious illness is of critical importance in febrile children who attend the emergency department (ED). Correct identification enables timely treatment of children with serious bacterial infections (SBIs) and children in need of intensive care unit (ICU) admission which improves patient outcomes.^1–4^ A recent review has studied the Shock Index, heart rate divided by systolic blood pressure (BP), as haemodynamic marker to predict disease severity in children and adults at the ED.^5^ Shock Index in adults has been studied in specific disease groups including trauma and myocardial infarction, and in a large general ED study in which high Shock Index >1.3 at triage has been associated with hospital admission and in-hospital mortality.^6^ In paediatrics, evidence of the Shock Index is limited to children with trauma,^7–10^ children with septic shock^11–13^ and a single-centre general ED population.^14^ To our knowledge, the Shock Index as a potential non-invasive measure in the early assessment for recognition of serious illness, including need for immediate life-saving interventions (ILSIs) and SBI, has not yet been evaluated. In addition, the association of the Shock Index with ICU admission in febrile children in a multicentre cohort is still unknown.

Like other vital signs, the normal ranges of the Shock Index are age dependent. Population-based centiles for Shock Index have been published for healthy children >8 years.^15^ Since fever increases heart rate values, reference values based on healthy children may not be generalisable to acutely ill children with fever attending the ED.^16 17^ In order to facilitate interpretation for clinical practice, clinical cut-off values are needed to classify children with high Shock Index.

We aimed (1) to derive reference values for the Shock Index based on this large ED population and (2) to determine the diagnostic value of the Shock Index for serious illness in febrile children attending European EDs.

## Methods

### Study design

This is a secondary analysis of the MOFICHE Study (Management and Outcome of Febrile children in Europe), embedded in the PERFORM Project (Personalized Risk assessment in Febrile illness to Optimize Real-life Management across the European Union).^18^ The MOFICHE Study is an observational multicentre study assessing the management and outcome of febrile children in Europe using routine data. Details of the study design are described previously.^19^

In short, children from 0 to 18 years presenting with fever (temperature ≥38.0°C) or with fever <72 hours before ED visit were included. Twelve EDs from eight European countries participated as part of the PERFORM Project: Austria, Germany, Greece, Latvia, the Netherlands (n=3), Spain, Slovenia and the UK (n=3). The participating hospitals were either university (n=9) or large teaching hospitals (n=3), and all were partners of the PERFORM consortium. Data were collected from January 2017 until April 2018 for at least 1 year. For the current study, we selected patients with routine BP measurement at the ED. For one ED (London, UK), BP measurements were not available and all visits from this ED were excluded.

Data collected were part of routine care and included sex, mode of referral (self-referral, general practitioner, private paediatrician, emergency medical services or other), comorbidity (chronic condition expected to last ≥1 year),^20^ alarming signs from the National Institute for Health and Care Excellence guideline on fever^21^ including consciousness (alert, voice, pain, unresponsive) and ill appearance as assessed by the physician, and vital signs: first measurement of temperature, heart rate, non-invasive systolic BP, capillary refill time. Heart rate was measured by pulse oximeters and systolic BP using oscillometric devices. In addition, we collected diagnostics (C reactive protein value (CRP) and blood cultures, cerebral spinal fluid cultures and other cultures) collected at the ED or first day of hospital admission. Further, we collected treatment with ILSI at the ED, defined as airway and breathing support (non-rebreathing mask, (non-invasive) ventilation, intubation), emergency procedures (chest needle decompression, pericardiocentesis or open thoracotomy), haemodynamic support (fluid bolus (>10 mL/kg) or blood administration) or emergency medication (naloxone, dextrose, atropine, adenosine, epinephrine or vasopressors).^22^ In addition, we collected data of prescribed antibiotics and general ward admission >24 hours, or ICU admission following ED visit.

To classify cause of infection in routine ED practice, we used a consensus-based flow chart^19^ combining all clinical data and diagnostic results. We used this flow chart to define the presumed cause of infection for each patient (online supplemental appendix 1). The diagnosis ‘definite bacterial’ infection was assigned when pathogenic bacteria were identified by sterile site culture or PCR. Patients were defined as ‘probable bacterial’ when a bacterial syndrome was suspected, but no bacteria were identified and CRP level was above 60 mg/L.^23^

10.1136/archdischild-2020-320992.supp1 Supplementary data

### Outcome measures

Serious illness was defined using four different outcomes: SBI, invasive bacterial infection (IBI), ILSI and all visits requiring ICU admission. Definition of SBI was decided on in a consensus meeting of experts in paediatrics and paediatric infectious disease specialists (PERFORM partners). SBI was defined as patients with ‘definite bacterial’ or ‘probable bacterial’ with focus on infection from the gastrointestinal tract, lower respiratory tract, urinary tract, bone and joints, central nervous system or sepsis.^24 25^ IBI, a subset of SBI, was defined as positive bacterial culture or PCR detection of a single pathogenic bacterium in blood, cerebrospinal fluid or synovial fluid. All cultures that were treated as contaminant and cultures growing contaminants were considered non-IBI.^26^ In addition, cultures growing a single contaminant or candida were defined positive in patients with malignancy, immunodeficiency, immunosuppressive drugs or a central catheter, since antimicrobial treatment is recommended in these patient groups.^27^

### Data analysis

We described the study population, and compared patients with and without BP measurement and focused the analysis on patients with BP measurement.

#### Part 1: Shock Index reference values

For the analysis on reference values, we excluded patients with immediate triage urgency as these patients are vitally compromised, and excluded children with missing heart rate values. First, we visualised heart rate and systolic BP by age using scatterplots. Second, we assessed the relation between heart rate and systolic BP using standardised z-scores calculated separately for different age groups: patients >1 year were grouped in 1-year age groups and patients <1 year were grouped in <3 months, 3–6 months and 6 months–1 year. Next, we calculated the Shock Index by dividing heart rate by systolic BP and calculated 95th centile Shock Index values in the different age groups.

#### Part 2: diagnostic value of Shock Index for serious illness

We evaluated the diagnostic value of the Shock Index using the following analyses: (1) the additional value of the Shock Index over systolic BP alone, (2) diagnostic performance of Shock Index above the 95th centile for each of the outcomes, and (3) stratified for age, we explored age-appropriate cut-off values of Shock Index for the different outcomes.

First, we assessed the additional value of the Shock Index to systolic BP by comparing a model with solely systolic BP to a model with both Shock Index and systolic BP (likelihood ratio test). Second, we used univariable logistic regression analysis to assess the association of Shock Index above the 95th centile with each of the outcomes. In multivariable analyses, we adjusted for age, sex, referral (referred vs self-referred), comorbidity and temperature. A previous study recommends to adjust for age besides the use of age-adjusted vital signs.^28^ Next, we calculated the diagnostic performance of Shock Index above the 95th centile for each of the outcomes using sensitivity, specificity, and negative and positive likelihood ratios (LRs). Negative LR <0.2 or positive LR >5 was defined as relevant.^29^ Furthermore, we described the ‘number needed to detect a disease’ which reflects the number needed to be examined in order to accurately detect on a person with the disease.^30^ Next, the discriminative ability of the Shock Index as continuous predictor for the outcomes was presented by area under the curve of receiver operating characteristics (AUROC) in different age groups. We used the following age groups to ensure sufficient numbers of the different outcomes for analysis: <1 year, 1–5 years, 5–10 years and >10 years. We explored age-appropriate cut-off values of the Shock Index for the different outcomes with a high sensitivity. We determined the optimal cut-off as a sensitivity of at least 90% with maximum specificity.

#### Missing values

Patients with missing data for the outcomes (cause of infection, focus of infection, ICU admission) were excluded from analysis (n=26). Missing values for referral, comorbidity, temperature, heart rate, capillary refill time and consciousness were multiple imputed including all available information of the patients using the mice package^31^ which resulted in 20 imputation sets (details in online supplemental appendix 2). In a sensitivity analysis, using a different approach to deal with missing BP data, we selected all EDs with >20% BP measurements and imputed missing BP values. In this subset, we repeated all analyses from part 2. All data analyses were performed in R V.3.6.

## Results

### Study population

**Figure 1:**
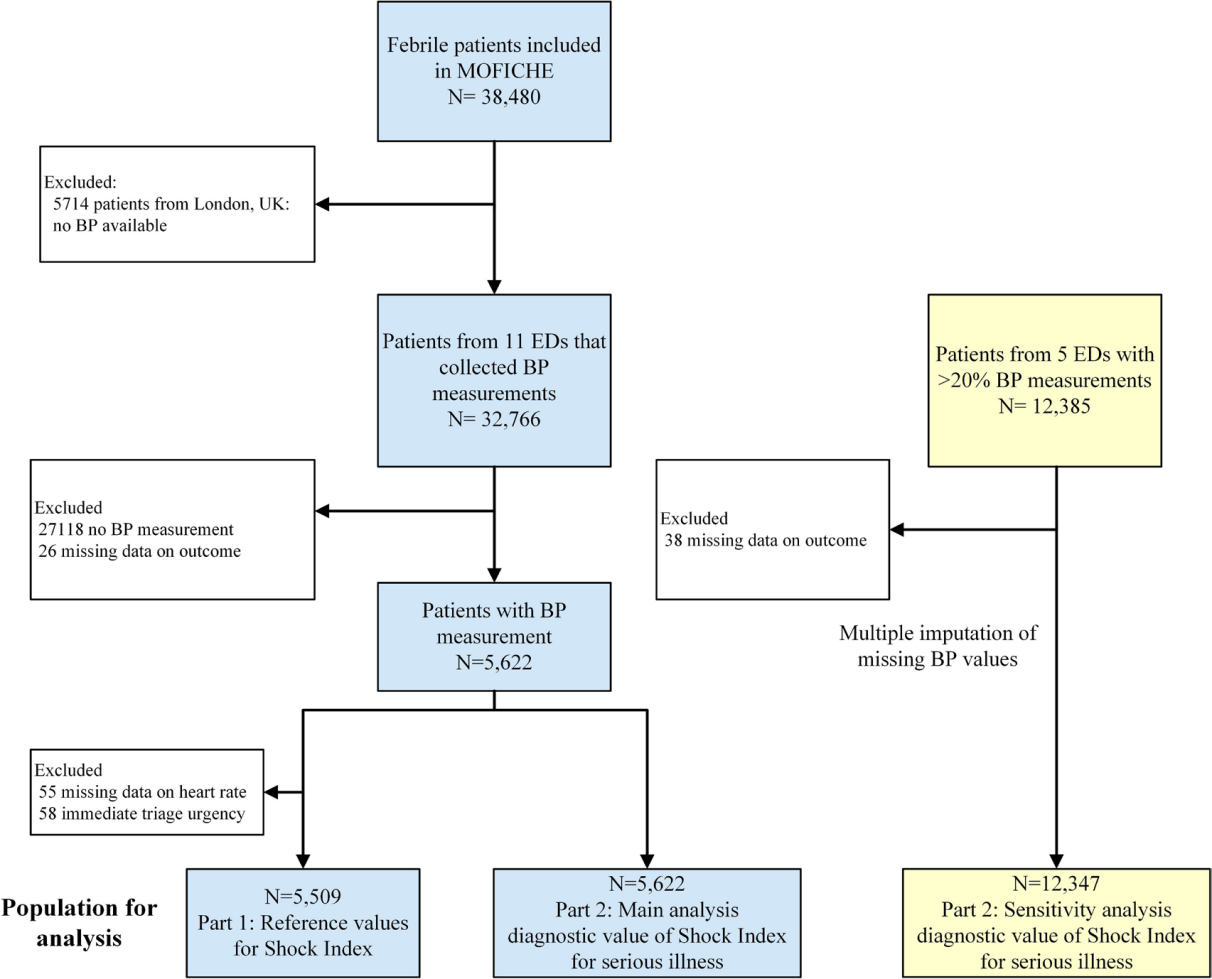
Flow chart of study population. BP, blood pressure; EDs, emergency departments.

Of 32 766 eligible patients, we included 5622 patients with BP measurement and complete outcome (2548 female (45.3%), median age 4.2 years (IQR 1.8–8.4)) (figure 1). Of those, 1338 (23.8%) patients had comorbidity and 2354 patients (41.9%) were referred to the ED. Regarding the outcomes, 461 patients (8.2%) had SBI, 46 (0.8) IBI, 203 (3.6%) patients were treated with ILSI and 69 (1.2%) were admitted to the ICU (table 1, details in online supplemental appendix 3). Of the 203 patients with ILSI, 30 (17.8%) were admitted to the ICU. Patients with BP measurement had more often one of the outcomes of serious illness than patients without BP measurement (details in online supplemental appendix 4).

**Table 1:** Clinical characteristics of the study population and for the different outcomes

	Study population, n=5622	Missing	SBI, n=461	IBI, n=46	ILSI, n=203	ICU admission, n=69
	n (%)	n	n (%)	n (%)	n (%)	n (%)
General characteristics						
Age in years, median (IQR)	4.2 (1.8–8.5)		5.3 (1.8–12.0)	4.8 (1.3–9.1)	4.1 (1.5–9.2)	2.8 (1.1–5.8)
Female	2548 (45.3)		228 (49.5)	21 (45.7)	89 (43.8)	36 (52.2)
Comorbidity	1338 (23.8)	91	167 (36.2)	29 (63.0)	92 (45.3)	28 (40.6)
Complex comorbidity	530 (9.4)		85 (18.4)	21 (45.7)	53 (26.1)	20 (29.0)
Referred	2354 (41.9)	110	293 (63.6)	35 (76.1)	152 (74.9)	55 (79.7)
Triage urgency		264				
Low: standard, non-urgent	1746 (31.1)		184 (39.9)	6 (13.0)	23 (11.3)	5 (7.3)
High: immediate, very urgent, intermediate	3612 (64.2)		224 (48.6)	37 (80.4)	159 (78.3)	58 (84.1)
Clinical symptoms						
Fever duration in days, median (IQR)	1.5 (0.5–3)	704	1.5 (0.5–3)	0.5 (0.5–3)	0.5 (0.5–1.5)	0.5 (0.5–1.5)
Ill appearance	868 (15.4)	620	173 (37.5)	22 (47.8)	106 (52.2)	40 (58.0)
Decreased consciousness	82 (1.5)	90	10 (2.2)	5 (10.9)	42 (20.7)	23 (33.3)
Vital signs						
Temperature in °C, median (IQR)	37.6 (36.8–38.4)	480	37.9 (37.1–38.7)	38.4 (37.7–39.2)	38.2 (37.3–39)	38.1 (37.1–38.7)
Prolonged capillary refill (>3 s)	105 (1.9)	866	24 (5.2)	3 (6.5)	39 (19.2)	18 (26.1)
Tachycardia (APLS)	1667 (29.7)	55	199 (43.2)	27 (58.7)	113 (55.7)	38 (55.1)
Hypotension (APLS)	209 (3.7)		38 (8.2)	3 (6.5)	22 (10.8)	10 (14.5)
Shock Index, median (IQR)	1.2 (1.0–1.4)	55	1.2 (1.0–1.5)	1.3 (1.9–1.6)	1.3 (1.1–1.6)	1.4 (1.2–1.7)
Shock Index, >95th centile for age	310 (5.5)	55	44 (9.5)	6 (13.0)	29 (14.3)	8 (11.6)
Diagnostics and treatment						
C reactive protein in mg/L, median (IQR)	20 (5–61)	3378	91 (38–154)	58 (17–147)	20 (5–75)	19 (4–83)
Blood cultures performed	967 (17.2)		243 (52.7)	46 (100)	118 (58.1)	44 (63.8)
Cerebrospinal fluid performed	140 (2.5)		34 (7.4)	8 (17.4)	28 (13.8)	20 (29.0)
Admission to the ward >24 hours	1159 (20.6)	137	281 (61.0)	34 (73.9)	109 (53.7)	
Admission to the ICU	69 (1.2)		19 (4.1)	7 (15.2)	43 (21.2)	69 (100)
Antibiotic treatment following ED visit	1983 (35.3)	55	407 (88.3)	44 (95.7)	151 (74.4)	50 (72.5)

APLS, advanced paediatric life support; ED, emergency department; IBI, invasive bacterial infection; ICU, intensive care unit; ILSI, immediate life-saving intervention; SBI, serious bacterial infection.

#### Part 1: Shock Index reference values

**Figure 2:**
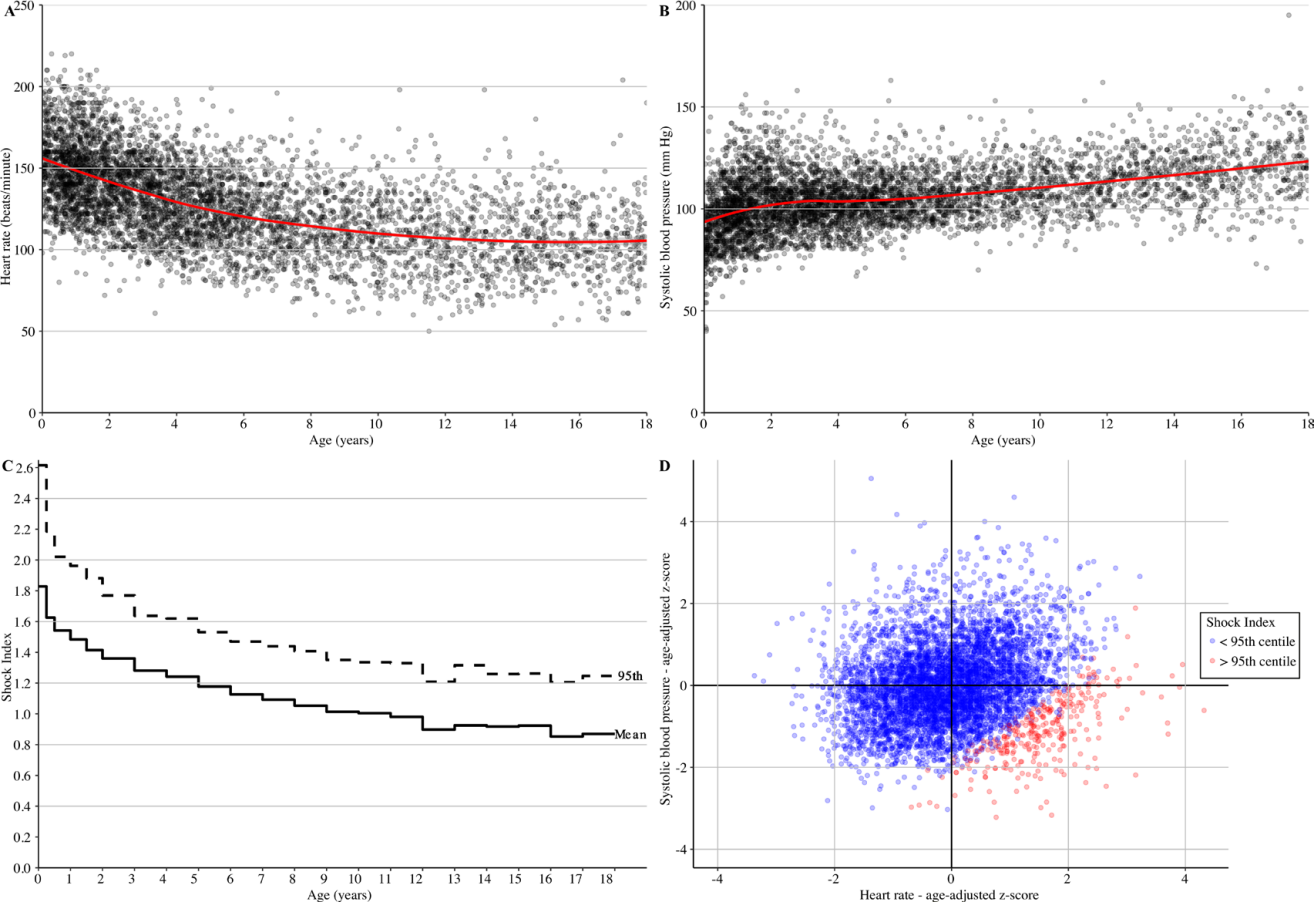
(A) Scatterplots of heart rate for age; (B) systolic blood pressure (BP) for age; (C) step chart of reference values of Shock Index (mean and 95th centile); (D) scatterplot of age-adjusted z-scores of systolic BP for age-adjusted z-scores of heart rate.

In our cohort of febrile children, systolic BP values increased with age, whereas heart rate and Shock Index values decreased with age (figure 2A–D, online supplemental appendix 5). The 95th centile for Shock Index was 2.61 for children <3 months and decreased to 1.21 for children aged 17–18 years. Overall, Shock Index values were higher in children with tachycardia or hypotension than in children without tachycardia or hypotension (p<0.001). Children with tachycardia or hypotension more often had Shock Index values above the 95th centile (293 of 1765, 16.6%) than children without tachycardia or hypotension (14 of 3744, 0.4%).

#### Part 2: diagnostic value of Shock Index for serious illness

Overall, 5.5% (310 of 5622) of patients had Shock Index values >95th centile. In patients with SBI, IBI, ILSI or ICU admission, high Shock Index >95th centile occurred in 9.5% (44 of 461), 13.0% (6 of 46), 14.3% (29 of 203) and 11.6% (8 of 69), respectively (table 1).

Addition of Shock Index to the model with only systolic BP led to a significant improved model for each of the outcomes (p<0.05). As a sole predictor, the 95th centile cut-off of Shock Index was associated with SBI (OR 1.9 (95% CI 1.6 to 2.3)), IBI (OR 2.6 (95% CI 1.7 to 3.4)), ILSI (OR 3.1 (95% CI 2.7 to 3.5)) and ICU admission (OR 2.6 (95% CI 1.9 to 3.3)). For SBI, ILSI and ICU admission, this association remained after adjustment for age, sex, referral, comorbidity and temperature (SBI: adjusted OR (aOR) 1.6 (95% CI 1.3 to 1.9); ILSI: aOR 2.5 (95% CI 2.0 to 2.9); ICU admission: aOR 2.2 (95% CI 1.4 to 2.9)), but the association was not significant for IBI (aOR 1.5 (95% CI 0.6 to 2.4)). The 95th centile cut-off of Shock Index had high specificity (all outcomes 0.95 (95% CI 0.94 to 0.95)) and positive LRs ranging from 1.8 to 2.8, but had low sensitivity (range 0.10–0.15) and poor negative LRs (range 0.90–0.95) for the different outcomes (table 2). The number needed to detect a disease for the 95th centile cut-off of Shock Index ranged from 10 to 20 for the different outcomes (table 2). Stratified by age, the AUROC of the Shock Index as continuous predictor ranged 0.55–0.66 for SBI, ranged 0.56–0.74 for IBI, ranged 0.57–0.71 for ILSI, and ranged 0.52–0.73 for ICU admission (table 3). Consequently, when attempting to define age-specific cut-off values, these had high sensitivity (>90%) but low specificity (0%–54%) for the different outcomes (online supplemental appendix 6).

**Table 2:** Diagnostic value of high Shock Index >95th centile for serious illness, n=5622

	OR (95% CI)	aOR* (95% CI)	Sensitivity (95% CI)	Specificity (95% CI)	Positive LR (95% CI)	Negative LR (95% CI)	Number needed to detect a disease (N)
SBI, n=461	1.9 (1.6 to 2.3)	1.6 (1.3 to 1.9)	0.10 (0.07 to 0.13)	0.95 (0.94 to 0.95)	1.8 (1.4 to 2.5)	0.95 (0.93 to 0.98)	20
IBI, n=46	2.6 (1.7 to 3.4)	1.5 (0.6 to 2.4)	0.13 (0.05 to 0.26)	0.95 (0.94 to 0.95)	2.4 (1.1 to 5.1)	0.92 (0.82 to 1.03)	12.5
ILSI, n=203	3.1 (2.7 to 3.5)	2.5 (2.0 to 2.9)	0.15 (0.10 to 0.20)	0.95 (0.94 to 0.95)	2.8 (2.0 to 4.0)	0.90 (0.85 to 0.95)	10
ICU admission, n=69	2.6 (1.9 to 3.3)	2.2 (1.4 to 2.9)	0.13 (0.06 to 0.23)	0.95 (0.94 to 0.95)	2.4 (1.3 to 4.5)	0.92 (0.84 to 1.01)	12.5

*Adjusted for age, sex, referral, comorbidity and temperature.

aOR, adjusted OR; IBI, invasive bacterial infection; ICU, intensive care unit; ILSI, immediate life-saving intervention; LR, likelihood ratio; SBI, serious bacterial infection.

**Table 3:** Discriminative value of Shock Index (continuous) for serious illness, stratified for age n=5622

	SBI	IBI	ILSI	ICU admission
	AUC (95% CI)	AUC (95% CI)	AUC (95% CI)	AUC (95% CI)
Shock Index (continuous) stratified for age				
<1 year, n=801	0.66 (0.60 to 0.72)	0.71 (0.56 to 0.85)	0.70 (0.60 to 0.80)	0.73 (0.59 to 0.87)
1–5 years, n=2395	0.54 (0.49 to 0.59)	0.56 (0.42 to 0.70)	0.57 (0.51 to 0.64)	0.58 (0.47 to 0.68)
5–10 years, n=1330	0.56 (0.50 to 0.62)	0.68 (0.50 to 0.86)	0.61 (0.52 to 0.69)	0.52 (0.36 to 0.69)
>10 years, n=1096	0.55 (0.50 to 0.60)	0.74 (0.63 to 0.85)	0.71 (0.64 to 0.79)	0.72 (0.45 to 0.98)

AUC, area under the curve; IBI, invasive bacterial infection; ICU, intensive care unit; ILSI, immediate life-saving intervention; SBI, serious bacterial infection.

The sensitivity analysis including all visits from the five EDs with >20% BP measurements (n=12 347) provided similar results for the diagnostic value of Shock Index >95th centile (online supplemental appendix 7).

## Discussion

In this large European multicentre study, we provided reference values for Shock Index in febrile children attending the ED. In addition, we evaluated the diagnostic value of Shock Index for serious illness defined as SBI, IBI, ILSI and ICU admission. High Shock Index showed an association with serious illness, but its rule-out value was poor.

Tachycardia and delayed capillary refill are early haemodynamic markers of shock, while hypotension is considered a late sign. The Shock Index combines the properties of heart rate and systolic BP and could potentially improve identification of acutely ill children at the ED. Previous studies in paediatrics have been studying the role of Shock Index in trauma, septic shock, and hospital and ICU admission.^5 7 8 10–14 32 33^ In our previous single-centre study, we found an association of high Shock Index for hospital and ICU admission in children with different presentations at the ED.^14^ Although this previous study included both febrile and non-febrile children, our study confirms an association of high Shock Index with SBI, ILSI and ICU admission in febrile children.

In adults, Shock Index values of >0.9 are related to hospital admission and mortality.^5 6^ In children, reference values and accurate cut-off values for Shock Index are yet unclear. Rappaport *et al* 15 have provided reference values of the Shock Index for healthy subjects aged >8 years based on auscultatory BP measurements. Gupta and Alam^13^ reported Shock Index values in a small study of children with sepsis for the outcome mortality. In this study, we provide reference values of the Shock Index for febrile children attending EDs. These values could be used as a reference value for clinical practice or further studies, although generalisability of these values to all febrile children or other populations may be limited.

In our sample of patients with measured BP, Shock Index values above the 95th centile cut-off value were associated with SBI, ILSI and ICU admission adjusted for age, sex, referral, comorbidity and temperature. In this multivariate analysis, Shock Index 95th centile was not significantly associated with IBI although the trend was similar. High Shock Index had high specificity and moderate positive LRs, but had poor rule-out value with low sensitivity and poor negative LRs. Its poor rule-out value makes the Shock Index not a valuable screening tool at the ED. Although we identified age-specific cut-off values with high sensitivity, none had adequate specificity and therefore leading to high number of false positives. Although this was not the focus of our study, the Shock Index may have additional value in specific high-risk patients or as repeated measurement for monitoring disease course or treatment effect.

Physiologically based scores have been developed for the early recognition of disease severity in children including scores as quick Sequential Organ Failure Assessment (qSOFA), quick Paediatric Logistic Organ Dysfunction-2 (qPELOD-2) and Liverpool qSOFA (LqSOFA).^34–37^ In previous ED studies, these scores showed high specificity but low sensitivity for serious illness.^36 37^ LqSOFA is based on heart rate and capillary refill time as haemodynamic parameters, whereas qSOFA and qPELOD-2 both require BP measurement. Since heart rate and capillary refill time are easy to assess in children, LqSOFA could be more easily implemented than scores that need BP measurement. The low sensitivity of these scores, however, makes them of limited clinical value for routine use at the ED.

Systolic BP measurement is also required for the Shock Index. The National Institute for Health and Care Excellence does not advise routine BP measurement in febrile children attending the ED,^21^ but recommends BP measurement in children with abnormal heart rate or prolonged capillary refill. In our cohort, BP measurement was performed in 1799 of 7804 (23%) of children with abnormal heart rate or capillary refill. This poor adherence to recommendations agrees with findings of moderate adherence to other vital sign measurements in febrile children in different European EDs.^38^

Strengths of this study include the participation of different EDs in Europe, the detailed data collection and the evaluation of the Shock Index for different definitions of serious illness: SBI, IBI, ILSI and ICU admittance, and adjustment for age, sex, referral, comorbidity and temperature. Our study has limitations. First, the selection of patients with BP measurement could have led to selection bias. Due to the limited number of BP measurements in our cohort, multiple imputation of systolic BP in all patients was not possible. In a sensitivity analysis, we imputed systolic BP in all visits of febrile children at the five EDs with >20% BP measurement and found similar results. This suggests that the selection of patients with BP measurement did not influence our results. The low proportion of BP measurement in our study reflects clinical practice where guidelines do not advise routine BP measurement in febrile children.^21 38^ Patients with BP measurement, however, likely reflect the group in which the Shock Index would potentially be used in clinical practice.

Second, we focused our analysis on high Shock Index since in febrile children we expect the combination of tachycardia and hypotension to be valuable. However, we recognise that hypotension without compensatory high heart rate is a relevant sign of shock which could result in normal Shock Index values. Lastly, the presence of hypotension or tachycardia may have influenced decisions to initiate treatment with ILSI or paediatric ICU admission. We acknowledge that Shock Index might not be a complete independent variable for these outcomes.

### Conclusions

In this large observational study of 11 European EDs, we provide reference values for Shock Index for febrile children at the ED. High Shock Index was associated with serious illness like SBI, IBI, ILSI and ICU admission. For serious illness, the rule-out value of high Shock Index was not sufficient. Our results suggest that the Shock Index is not valuable as a routine screening tool in the early assessment of febrile children at the ED.

## Data Availability

Data are available in a public, open access repository. A data set containing individual participant data will be made available in a public data repository containing a specific DOI. The data will be anonymised and will not contain any identifiable data. The data manager of the PERFORM consortium can be contacted for inquiries (Tisham.de@imperial.ac.uk).
