# Supplementary material for: Shock Index in the early assessment of febrile children at the emergency department: a prospective multicentre study
Source: Arch Dis Child. 2021 Jun 22;107(2):116–22. doi: 10.1136/archdischild-2020-320992 (PMC8784994; doi:10.1136/archdischild-2020-320992)
Supplement: Supplementary data [file archdischild-2020-320992supp001.pdf]

Appendix 1: Flowchart to classify presumed cause of infection

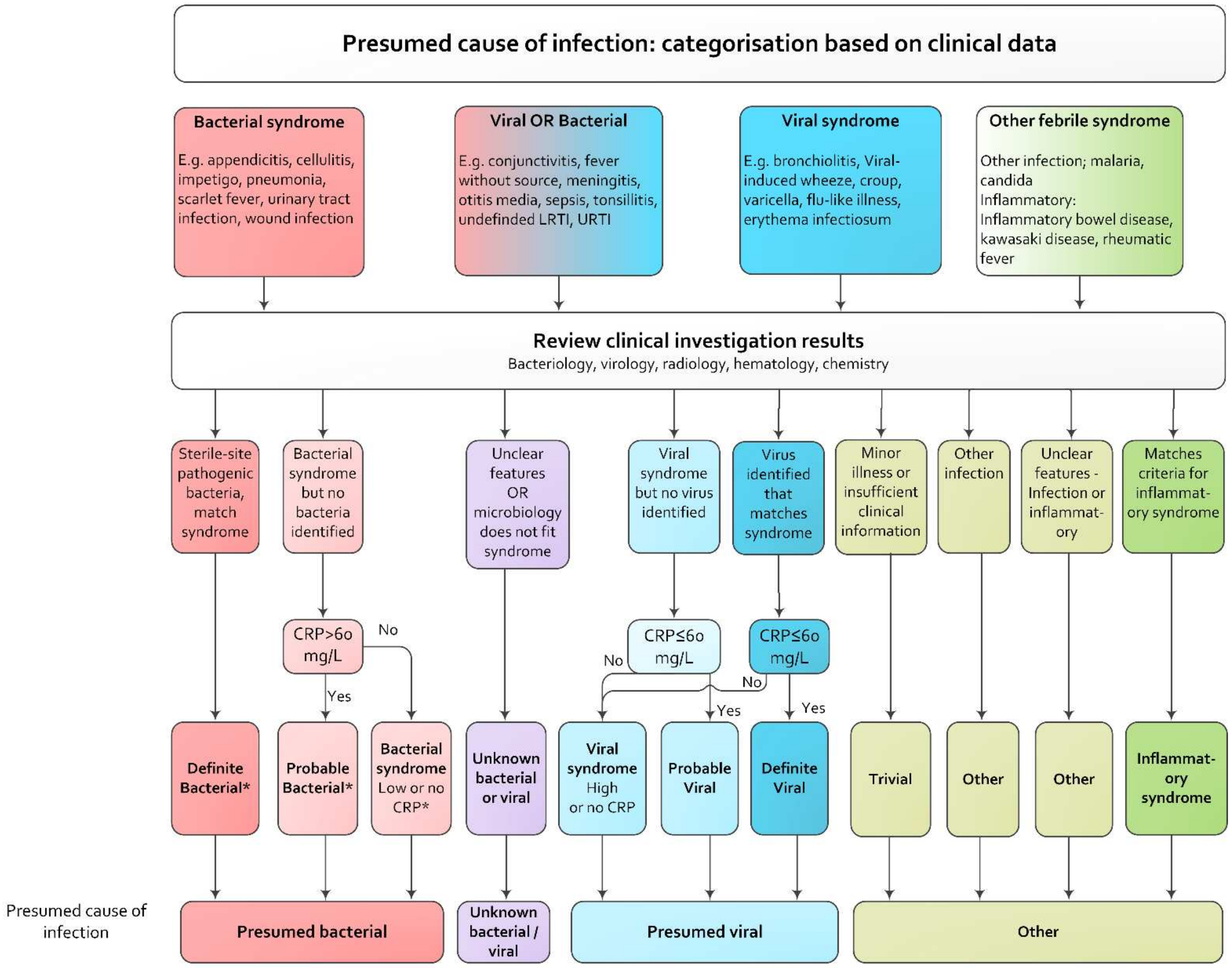

CRP, c-reactive protein; LRTI, lower respiratory tract infection; URTI, upper respiratory tract infection.
*Patients could have identified viral co-infection. (1)

References

1. Hagedoorn NN, Borensztajn DM, Nijman R, et al. Variation in antibiotic prescription rates in febrile children presenting to emergency departments across Europe (MOFICHE): A multicentre observational study. *PLOS Medicine*. 2020;17:e1003208.

Appendix 2. Additional methods: multiple imputation

Missing data

For the main analysis, we excluded patients without systolic blood pressure (BP) measurement. We used multiple imputation by chained equations using the MICE package in R to impute referral, comorbidity, temperature, heart rate, capillary refill time and consciousness. We included hospital, all outcome measures and other auxiliary variables influencing case-mix and disease severity in the imputation model. Multiple imputation was performed on all patients (n=32,766). For the statistical analysis where we used the multiple imputation data, results were pooled for a final result. For the main analysis, patients with missing systolic BP measurement were excluded leading to 5648 eligible visits.

For the sensitivity analysis, we used a different approach to deal with missing BP data. We selected the five EDs with >20% BP measurements (n=12,385), and imputed missing BP values in this subset. In this subset we repeated all analysis from part 2.
Proportion of missingness of variables are provided in Table 1 and Appendix 5.

Variables in the multiple imputation model:

General characteristics	Markers of disease severity	Vital signs	Diagnostics	Treatment	Outcomes
Hospital	Triage urgency	Heart rate	CRP-level	Immediate life-saving interventions	Disposition
Age	Fever duration	Respiratory rate	Chest X-ray categories	Oxygen treatment	Final diagnosis
Sex	Ill appearance	Temperature	Urinalysis categories	Inhalation medication	Focus of infection
Referral type (self / GP / emergency services / other)	Work of breathing	Capillary refill time	Blood culture performed	Antibiotic prescription type	Serious bacterial infection
Previous medical care (yes, primary care / yes, this ED / yes other secondary care)	Consciousness	Oxygen saturation	Cerebrospinal fluid performed	Antibiotic prescription mode	Invasive bacterial infection
Season	Meningeal signs	Non-invasive systolic blood pressure			
Comorbidity	Focal neurology				
Complex comorbidity	Non-blanching rash				
	Dehydration				
	Seizures				

Appendix 3. Further details of serious bacterial infections (n=461), invasive bacterial infections (n=46) and immediate-lifesaving interventions (n=203)

**Infection focus of serious bacterial infections (n=461)**	**N (%)**
Urinary tract	153 (33.2%)
Lower respiratory tract infection	139 (30.2%)
Gastro intestinal or surgical abdomen	93 (20.2%)
Sepsis	37 (8.0%)
Musculoskeletal	15 (3.3%)
Meningitis / CNS infection	10 (2.2%)
Other	14 (3.0%)

**Invasive bacterial infections (n=46)**	**N (%)**
Bacteraemia*	40 (87%)
Bacterial meningitis*	6 (13%)
Bone and joint	2 (4.3%)

*Two patients had both bacteraemia and bacterial meningitis

**Immediate life-saving interventions (n=203)***	**N (%)**
Airway/breathing interventions	100 (49.3%)
Haemodynamic interventions	112 (55.2%)
Emergency medications	52 (26.6%)

*Multiple categories per patients possible

Appendix 4. Patient characteristics of patients with blood pressure measurement and patients without blood pressure measurement

	Blood pressure measured (n=5622)		No blood pressure measured (n=26841)	
	**n (%)**	**Missing**	**n (%)**	**Missing**
**General characteristics**				
Age in years, median (IQR)	4.2 (1.8-8.5)		2.6 (1.3-5.2)	
Female	2548 (45.3)		12172 (45.3)	
Comorbidity	1338 (23.8)	91	3831 (14.3)	182
Complex comorbidity	530 (9.4)	91	931 (3.5)	182
Referred	2354 (41.9)	110	11028 (41.1)	1044
Triage urgency		264		879
Low: standard, non-urgent	3612 (64.2)		18670 (69.6)	
High: immediate, very urgent, intermediate	1746 (31.1)		7292 (27.2)	
**Clinical symptoms**				
Fever duration in days, median (IQR)	1.5 (0.5-3)	704	1.5 (0.5-3)	1676
Ill appearance	868 (15.4)	621	4855 (18.1)	1040
Decreased consciousness	82 (1.5)	90	87 (0.3)	210
**Vital signs**				
Temperature in °C, median (IQR)	37.6 (36.8-38.4)	480	37.7 (37.0-38.4)	2432
Hypoxia <95%	2920(5.2)	211	935 (3.5)	5204
Prolonged capillary refill (>3 sec)	105 (1.9)	866	254 (0.9)	3004
Tachycardia (APLS)	1667 (29.7)	55	5537 (20.6)	3372
**Diagnostics and treatment**				
CRP in mg/L, median (IQR)	20 (5-61)	3378	17 (5-47)	13021
Blood cultures performed	967 (17.2)		1798 (6.7)	
Cerebrospinal fluid performed	140 (2.5)		198 (0.7)	
Antibiotic treatment following ED visit	1983 (35.2)	55	8305 (30.9)	398
Admission to the ward >24 hours	1159 (20.6)	137	5415 (20.2)	328
**Serious illness**				
Serious bacterial infection	461 (8.2)		1683 (6.3)	
Invasive bacterial infection	46 (0.8)		82 (0.3)	
Admission to the ICU	69 (1.2)		76 (0.3)	
Immediate life-saving interventions	203 (3.6)		212 (0.8)	

APLS, advanced paediatric life support; CRP, C-reactive protein; ICU, intensive care unit; IQR, interquartile range; NA, not applicable

Appendix 5. Shock Index reference values according to age, n=5509

Age group	N	Shock Index Mean (SD)	Shock Index 95th centile
<3m	181	1.83 (0.48)	2.62
3-6m	163	1.63 (0.34)	2.19
6m-1y	430	1.54 (0.29)	2.02
1-2y	753	1.45 (0.29)	1.96
2-3y	574	1.36 (0.25)	1.88
3-4y	549	1.28 (0.22)	1.77
4-5y	462	1.24 (0.23)	1.64
5-6y	406	1.18 (0.21)	1.62
6-7y	276	1.13 (0.21)	1.53
7-8y	234	1.09 (0.21)	1.47
8-9y	196	1.05 (0.22)	1.44
9-10y	185	1.01 (0.20)	1.41
10-11y	166	1.00 (0.20)	1.35
11-12y	157	0.98 (0.21)	1.34
12-13y	139	0.90 (0.19)	1.33
13-14y	127	0.93 (0.24)	1.21
14-15y	159	0.92 (0.21)	1.32
15-16y	122	0.92 (0.21)	1.26
16-17y	99	0.85 (0.21)	1.26
17-18y	131	0.87 (0.23)	1.21

SD, standard deviation; m, months; y, year

Appendix 6. Shock Index cut-off values for the different outcomes, stratified for age groups

Serious bacterial infection	Shock Index cut-off value*	Sensitivity	Specificity	Negative LR	Positive LR
Age <1 year	1.37	0.91	0.24	0.37	1.20
Age 1-5 year	1.12	0.90	0.18	0.54	1.10
Age 5-10 year	0.81	0.91	0.08	1.21	0.98
Age >10 year	0.67	0.90	0.11	0.88	1.02

Invasive bacterial infection	Shock Index cut-off value*	Sensitivity	Specificity	Negative LR	Positive LR
Age <1 year	1.43	1.00	0.31	0.00	1.45
Age 1-5 year	1.19	0.92	0.29	0.29	1.28
Age 5-10 year	0.79	0.92	0.07	1.26	0.98
Age >10 year	0.93	0.91	0.54	0.17	1.98

Immediate life-saving intervention	Shock Index cut-off value*	Sensitivity	Specificity	Negative LR	Positive LR
Age <1 year	1.40	0.91	0.27	0.34	1.24
Age 1-5 year	1.06	0.91	0.12	0.78	1.03
Age 5-10 year	0.96	0.92	0.25	0.33	1.22
Age >10 year	0.79	0.92	0.29	0.29	1.29

ICU admission	Shock Index cut-off value*	Sensitivity	Specificity	Negative LR	Positive LR
Age <1 year	1.32	0.94	0.18	0.33	1.14
Age 1-5 year	1.11	0.90	0.17	0.56	1.09
Age 5-10 year	0.68	0.93	0.02	4.25	0.94
Age >10 year	0.53	1.00	0.01	0.00	1.01

* minimal sensitivity >=90% and maximal specificity

Appendix 7. Sensitivity analysis for febrile children in 5 EDs with >20% SBP measurement (n=12347)

Univariate and multivariate analysis of Shock Index >95th centile values for serious illness (n=12347)

	Shock Index >95th centile value	
	**OR (95% CI)**	**Adj. OR (95% CI)***
**SBI** n=643	1.7 (1.2-2.4)	1.4 (1.0-2.0)
**IBI** n=81	2.0 (0.8-4.8)	1.7 (0.7-4.1)
**ILSI** n=336	2.6 (1.8-3.8)	2.4 (1.6-3.6)
**ICU admission** n=90	2.9 (1.5-5.5)	3.0 (1.5-5.8)

*Adjusted for age, sex, referral (y/n), comorbidity (y/n), temperature
Adj, adjusted; CI, confidence interval; ICU, intensive care unit; OR, odds ratio

Diagnostic performance of high Shock Index >95th centile for serious illness (n=12347)

	Sensitivity (95% CI)	Specificity (95% CI)	Positive LR (95% CI)	Negative LR (95% CI)
**SBI**	0.08 (0.06-0.10)	0.97 (0.96-0.97)	2.4 (1.8-3.2)	0.95 (0.93-0.97)
**IBI**	0.10 (0.04-0.19)	0.97 (0.96-0.97)	2.9 (1.5-5.7)	0.93 (0.87-1.00)
**ILSI**	0.13 (0.09-0.17)	0.97 (0.96-0.97)	3.9 (2.9-5.3)	0.90 (0.87-0.94)
**ICU admission**	0.14 (0.08-0.23)	0.97 (0.96-0.97)	4.3 (2.6-7.20)	0.89 (0.81-0.96)

Discriminative value of Shock Index (continuous) for serious illness, stratified for age n=12347

	SBI AUC (95% CI)	IBI AUC (95% CI)	ILSI AUC (95% CI)	ICU admission AUC (95% CI)
**Shock Index (continuous) stratified for age**				
<1 year, n=2337	0.63 (0.57-0.68)	0.71 (0.58-0.84)	0.69 (0.61-0.77)	0.71 (0.59-0.83)
1-5 year, n=6064	0.55 (0.51-0.60)	0.56 (0.42-0.69)	0.59 (0.54-0.65)	0.57 (0.46-0.67)
5-10 year, n=2484	0.53 (0.46-0.59)	0.65 (0.50-0.81)	0.56 (0.48-0.64)	0.53 (0.36-0.69)
>10 year, n= 1462	0.59 (0.53-0.65)	0.63 (0.46-0.80)	0.66 (0.59-0.74)	0.73 (0.48-0.98)

AUC, area under the curve; CI, confidence interval; IBI, invasive bacterial infection; ICU, intensive care unit; ILSI, immediate life-saving intervention; SBI, serious bacterial infection

Appendix 8: Members of PERFORM Consortium V6.0

PERFORM Consortium

PARTNER: IMPERIAL COLLEGE (UK)

Chief investigator/PERFORM coordinator:

Michael Levin

Principal and co-investigators; work package leads (alphabetical order)

Aubrey Cunnington (grant application)

Tisham De (work package lead)

Jethro Herberg (Principle Investigator, Deputy Coordinator, grant application)

Myrsini Kaforou (grant application, work package lead)

Victoria Wright (grant application, Scientific Coordinator)

Research Group (alphabetical order)

Lucas Baumard; Evangelos Bellos; Giselle D'Souza; Rachel Galassini; Dominic Habgood-Coote; Shea Hamilton; Clive Hoggart; Sara Hourmat; Heather Jackson; Ian Maconochie; Stephanie Menikou; Naomi Lin; Samuel Nichols; Ruud Nijman; Ivonne Pena Paz; Priyen Shah; Ching-Fen Shen; Clare Wilson

Clinical recruitment at Imperial College Healthcare NHS Trust (alphabetical order))

Amina Abdulla; Ladan Ali; Sarah Darnell; Rikke Jorgensen; Sobia Mustafa; Salina Persand

Imperial College Faculty of Engineering

Molly Stevens (co-investigator), Eunjung Kim (research group); Benjamin Pierce (research group)

Clinical recruitment at Brighton and Sussex University Hospitals

Katy Fidler (Principle Investigator)

Julia Dudley (Clinical Research Registrar)

Research nurses: Vivien Richmond, Emma Tavliavini

Clinical recruitment at National Cheng Kung University Hospital

Ching-Fen Shen (Principal Investigator); Ching-Chuan Liu (Co-investigator); Shih-Min Wang (Co-investigator), funded by the Center of Clinical Medicine Research, National Cheng Kung University

SERGAS Partner (Spain)

Principal Investigators

Federico Martinón-Torres[1]

Antonio Salas[1,2]

GENVIP RESEARCH GROUP (in alphabetical order):

Fernando Álvez González[1], Cristina Balo Farto[1], Ruth Barral-Arca[1,2], María Barreiro Castro[1], Xabier Bello[1,2], Mirian Ben García[1], Sandra Carnota[1], Miriam Cebey-López[1], María José Curras-Tuala[1,2], Carlos Durán Suárez[1], Luisa García Vicente[1], Alberto Gómez-Carballa[1,2], Jose Gómez Rial[1], Pilar Leboráns Iglesias[1], Federico Martinón-Torres[1], Nazareth Martinón-Torres[1], José María Martinón Sánchez[1], Belén Mosquera Pérez[1], Jacobo Pardo-Seco[1,2], Lidia Piñeiro Rodríguez[1], Sara Pischedda[1,2], Sara Rey Vázquez[1], Irene Rivero Calle[1], Carmen Rodríguez-Tenreiro[1], Lorenzo Redondo-Collazo[1], Miguel Sadiki Ora[1], Antonio Salas[1,2], Sonia Serén Fernández[1], Cristina Serén Trasorras[1], Marisol Vilas Iglesias[1].

[1] Translational Pediatrics and Infectious Diseases, Pediatrics Department, Hospital Clínico Universitario de Santiago, Santiago de Compostela, Spain, and GENVIP Research Group (www.genvip.org), Instituto de Investigación Sanitaria de Santiago, Universidad de Santiago de Compostela, Galicia, Spain.

[2] Unidade de Xenética, Departamento de Anatomía Patolóxica e Ciencias Forenses, Instituto de Ciencias Forenses, Facultade de Medicina, Universidade de Santiago de Compostela, and GenPop Research Group, Instituto de Investigaciones Sanitarias (IDIS), Hospital Clínico Universitario de Santiago, Galicia, Spain

[3] Fundación Pública Galega de Medicina Xenómica, Servizo Galego de Saúde (SERGAS), Instituto de Investigaciones Sanitarias (IDIS), and Grupo de Medicina Xenómica, Centro de Investigación Biomédica en Red de Enfermedades Raras (CIBERER), Universidade de Santiago de Compostela (USC), Santiago de Compostela, Spain

RSU Partner (Latvia)

Principal Investigator

Dace Zavadska[1,2]

Other RSU group authors (in alphabetical order):

Anda Balode[1,2], Arta Bārzdiņa[1,2], Dārta Deksne[1,2], Dace Gardovska[1,2], Dagne Grāvele[2], Ilze Grope[1,2], Anija Meiere[1,2], Ieva Nokalna[1,2], Jana Pavāre[1,2], Zanda Pučuka[1,2], Katrīna Selecka[1,2], Aleksandra Sidorova[1,2], Dace Svile[2], Urzula Nora Urbāne[1,2].

[1] Riga Stradins university, Riga, Latvia.
[2] Children clinical university hospital, Riga, Latvia.

Medical Research Council Unit The Gambia (MRCG) at LSHTM Partner

Principal Investigator
Effua Usuf

Additional Investigators
Kalifa Bojang
Syed M. A. Zaman
Fatou Secka
Suzanne Anderson
Anna Rocalsatou Sarr
Momodou Saidykhan
Saffiatou Darboe
Samba Ceesay
Umberto D'alessandro

Medical Research Council Unit The Gambia at LSHTM
P O Box 273,
Fajara, The Gambia

ERASMUS MC-Sophia Children's Hospital

Principal Investigator
Henriëtte A. Moll[1]

Research group
Dorine M. Borensztajn[1], Nienke N. Hagedoorn, Chantal Tan [1], [1], Clementien L. Vermont[2], Joany Zachariasse [1]

Additional investigator
W Dik [3]

[1] Erasmus MC-Sophia Children's Hospital, Department of General Paediatrics, Rotterdam, the Netherlands
[2] Erasmus MC-Sophia Children's Hospital, Department of Paediatric Infectious Diseases & Immunology, Rotterdam, the Netherlands
[3] Erasmus MC, Department of immunology, Rotterdam, the Netherlands

Swiss Pediatric Sepsis Study

Principal Investigators*:*

Philipp Agyeman, MD [1], Luregn J Schlapbach, MD, FCICM [2,3], Eric Giannoni, MD [4,5], Martin Stocker, MD [6], Klara M Posfay-Barbe, MD [7], Ulrich Heininger, MD [8], Sara Bernhard-Stirnemann, MD [9], Anita Niederer-Loher, MD [10], Christian Kahlert, MD [10], Giancarlo Natalucci, MD [11], Christa Relly, MD [12], Thomas Riedel, MD [13], Christoph Aebi, MD [1], Christoph Berger, MD [12] **for the Swiss Pediatric Sepsis Study**

Affiliations:

[1] Department of Pediatrics, Inselspital, Bern University Hospital, University of Bern, Switzerland

[2] Neonatal and Pediatric Intensive Care Unit, Children's Research Center, University Children's Hospital Zurich, University of Zurich, Zurich, Switzerland

[3]Child Health Research Centre, University of Queensland, and Queensland Children`s Hospital, Brisbane, Australia

[4] Clinic of Neonatology, Department Mother-Woman-Child, Lausanne University Hospital and University of Lausanne, Switzerland

[5] Infectious Diseases Service, Department of Medicine, Lausanne University Hospital and University of Lausanne, Switzerland

[6] Department of Pediatrics, Children's Hospital Lucerne, Lucerne, Switzerland

[7] Pediatric Infectious Diseases Unit, Children's Hospital of Geneva, University Hospitals of Geneva, Geneva, Switzerland

[8] Infectious Diseases and Vaccinology, University of Basel Children's Hospital, Basel, Switzerland

[9] Children's Hospital Aarau, Aarau, Switzerland

[10] Division of Infectious Diseases and Hospital Epidemiology, Children's Hospital of Eastern Switzerland St. Gallen, St. Gallen, Switzerland

[11] Department of Neonatology, University Hospital Zurich, Zurich, Switzerland

[12] Division of Infectious Diseases and Hospital Epidemiology, and Children's Research Center, University Children's Hospital Zurich, Switzerland

[13] Children's Hospital Chur, Chur, Switzerland

Liverpool Partner

Principal Investigators

Enitan D Carrol[1,2,3]

Stéphane Paulus [1,]

Research Group (in alphabetical order):

Elizabeth Cocklin[1], Rebecca Jennings[4], Joanne Johnston[4], Simon Leigh[1], Karen Newall[4], Sam Romaine[1]

[1] Department of Clinical Infection, Microbiology and Immunology, University of Liverpool Institute of Infection and Global Health , Liverpool, England
[2] Alder Hey Children's Hospital, Department of Infectious Diseases, Eaton Road, Liverpool, L12 2AP
[3] Liverpool Health Partners, 1st Floor, Liverpool Science Park, 131 Mount Pleasant, Liverpool, L3 5TF

[4]Alder Hey Children's Hospital, Clinical Research Business Unit, Eaton Road, Liverpool, L12 2AP

NKUA Partner (Greece)

Principal investigator: Professor **Maria Tsolia** (all activities)

Investigator/Research fellow: **Irini Eleftheriou** (all activities)

Additional investigators:

Recruitment: Maria Tambouratzi

Lab: Antonis Marmarinos (Quality Manager)

Lab: Marietta Xagorari

Kelly Syggelou

2nd Department of Pediatrics, National and Kapodistrian University of Athens,

"P. and A. Kyriakou" Children's Hospital

Thivon and Levadias

Goudi, Athens

Micropathology Ltd :

Dr Marie Voice Post doc scientist

Professor Colin Fink , Clinical Microbiologist

Additional investigators

Dr Marie Voice, Post doc scientist

Dr. Leo Calvo-Bado, Post doc scientist

Medical University of Graz, Austria (MUG)

Principal Investigator:

Werner Zenz[1] (all activities)

Co-investigators (in alphabetical order)

Benno Kohlmaier[1] (all activities)

Nina A. Schweintzger[1] (all activities)

Manfred G. Sagmeister[1] (study design, consortium wide sample management)

Research team

Daniela S. Kohlfürst[1] (study design)

Christoph Zurl[1] (BIVA PIC)

Alexander Binder[1] (grant application)

Recruitment team, data managers, (in alphabetical order):

Susanne Hösele[1], Manuel Leitner[1], Lena Pölz[1], Glorija Rajic[1],

Clinical recruitment partners (in alphabetical order):

Sebastian Bauchinger[1], Hinrich Baumgart[4], Martin Benesch[3], Astrid Ceolotto[1], Ernst Eber[2], Siegfried Gallistl[1], Gunther Gores[5], Harald Haidl[1], Almuthe Hauer[1], Christa Hude[1], Markus Keldorfer[5], Larissa Krenn[4], Heidemarie Pilch[5], Andreas Pfleger[2], Klaus Pfurtscheller[4], Gudrun Nordberg[5], Tobias Niedrist[8], Siegfried Rödl[4], Andrea Skrabl-Baumgartner[1], Matthias Sperl[7], Laura Stampfer[5], Volker Strenger[3], Holger Till[6], Andreas Trobisch[5], Sabine Löffler[5]

Author Affiliations:

[1] Department of Pediatrics and Adolescent Medicine, Division of General Pediatrics, Medical University of Graz, Graz, Austria

[2]Department of Pediatric Pulmonology, Medical University of Graz, Graz, Austria

[3]Department of Pediatric Hematooncoloy, Medical University of Graz, Graz, Austria

[4]Paediatric Intensive Care Unit, Medical University of Graz, Graz, Austria

[5]University Clinic of Paediatrics and Adolescent Medicine Graz, Medical University Graz, Graz,Austria

[6]Department of Paediatric and Adolescence Surgery, Medical University Graz, Graz, Austria

[7]Department of Pediatric Orthopedics, Medical University Graz, Graz, Austria

[8]Clinical Institute of Medical and Chemical Laboratory Diagnostics, Medical University Graz, Graz, Austria

London School of Hygiene and Tropical Medicine

WP 5/WP1

Principal Investigator:

Dr Shunmay Yeung[1,2 3] PhD, MBBS, FRCPCH, MRCP, DTM&H

Research Group

Dr Juan Emmanuel Dewez[1] MD, DTM&H, MSc

Mr David Bath[2] MSc, MAppFin, BA(Hons)

Dr Alec Miners[2] BA(Hons), MSc, PhD

Dr Ruud Nijman[3] PhD MSc MD MRCPCH

Dr Catherine Wedderburn[1] BA, MBChB, DTM&H, MSc, MRCPCH

Ms Anne Meierford[1] MSc, BMedSc, BMBS

Dr Baptiste Leurent[4], PhD, MSc

1. Faculty of Infectious and Tropical Disease, London School of Hygiene and Tropical Medicine, London, UK
2. Faculty of Public Health and Policy, London School of Hygiene and Tropical Medicine, London, UK
3. Department of Paediatrics, St. Mary's Hospital Imperial College Hospital, London, UK
4. Faculty of Epidemiology and Population Health, London School of Hygiene and Tropical Medicine, London, UK

Radboud University Medical Center (RUMC), The Netherlands

Principal Investigators:
Ronald de Groot [1], Michiel van der Flier [1,2,3], Marien I. de Jonge[1]

Co-investigators Radboud University Medical Center (in alphabetical order):
Koen van Aerde[1,2], Wynand Alkema[1], Bryan van den Broek[1], Jolein Gloerich[1], Alain J. van Gool[1], Stefanie Henriet[1,2], Martijn Huijnen[1], Ria Philipsen[1], Esther Willems[1]

Investigators PeDBIG PERFORM DUTCH CLINICAL NETWORK (in alphabetical order):
G.P.J.M. Gerrits[8], M. van Leur[8,] J. Heidema [4],L. de Haan[1,2] C.J. Miedema [5], C. Neeleman [1] C.C. Obihara [6], G.A. Tramper-Stranders7[6]

1. Radboud University Medical Center, Nijmegen, The Netherlands
2. Amalia Children's Hospital, Nijmegen, The Netherlands
3. Wilhelmina Children's Hospital, University Medical Center Utrecht, Utrecht, The Netherlands
4. St. Antonius Hospital, Nieuwegein, The Netherlands
5. Catharina Hospital, Eindhoven, The Netherlands
6. ETZ Elisabeth, Tilburg, The Netherlands
7. Franciscus Gasthuis, Rotterdam, The Netherlands
8. Canisius Wilhelmina Hospital, Nijmegen, The Netherlands

Oxford team (UK)

Principal Investigators

Andrew J. Pollard[1,2], Rama Kandasamy[1,2], Stéphane Paulus [1,2]

Additional Investigators

Michael J. Carter[1,2], Daniel O'Connor[1,2], Sagida Bibi[1,2], Dominic F. Kelly[1,2], Meeru Gurung[3], Stephen Thorson[3], Imran Ansari[3], David R. Murdoch[4], Shrijana Shrestha[3].

Author Affiliations:

[1]Oxford Vaccine Group, Department of Paediatrics, University of Oxford, Oxford, United Kingdom.

[2]NIHR Oxford Biomedical Research Centre, Oxford, United Kingdom.

[3]Paediatric Research Unit, Patan Academy of Health Sciences, Kathmandu, Nepal.

[4]Department of Pathology, University of Otago, Christchurch, New Zealand.

Newcastle University, Newcastle upon Tyne, (UK)

Principal Investigator:

Marieke Emonts [1,2,3] (all activities)

Co-investigators

Emma Lim[2,3,7] (all activities)

Lucille Valentine[4]

Recruitment team (alphabetical), data-managers, and GNCH Research unit:

Karen Allen[5], Kathryn Bell[5], Adora Chan[5], Stephen Crulley[5], Kirsty Devine[5], Daniel Fabian[5], Sharon King[5], Paul McAlinden[5], Sam McDonald[5], Anne McDonnell2,[5], Ailsa Pickering[2,5], Evelyn Thomson[5], Amanda Wood[5], Diane Wallia[5], Phil Woodsford[5],

Sample processing: Frances Baxter[5], Ashley Bell[5], Mathew Rhodes[5]

PICU recruitment

Rachel Agbeko[8]

Christine Mackerness[8]

Students MOFICHE

Bryan Baas[2], Lieke Kloosterhuis[2], Wilma Oosthoek[2]

Students/medical staff PERFORM

Tasnim Araf[6], Joshua Bennet[2], Kalvin Collings[2], Ilona van der Giessen[2], Alex Martin[2], Aqeela Rashid[6], Emily Rowlands[2] Gabriella de Vries[2]

Engagement work/ethics/cost effectiveness

Mike Martin[4], Ravi Mistry[2], Lucille Valentine[4]

Author Affiliations:

[1] Translational and Clinical Research Institute, Newcastle University, Newcastle upon Tyne UK

[2]Great North Children's Hospital, Paediatric Immunology, Infectious Diseases & Allergy, Newcastle upon Tyne Hospitals NHS Foundation Trust, Newcastle upon Tyne, United Kingdom.

[3]NIHR Newcastle Biomedical Research Centre based at Newcastle upon Tyne Hospitals NHS Trust and Newcastle University, Westgate Rd, Newcastle upon Tyne NE4 5PL, United Kingdom

[4]Newcastle University Business School, Centre for Knowledge, Innovation, Technology and Enterprise (KITE), Newcastle upon Tyne, United Kingdom

[5]Great North Children's Hospital, Research Unit, Newcastle upon Tyne Hospitals NHS Foundation Trust, Newcastle upon Tyne, United Kingdom.

[6]Great North Children's Hospital, Paediatric Oncology, Newcastle upon Tyne Hospitals NHS Foundation Trust, Newcastle upon Tyne, United Kingdom.

[7]Population Health Sciences Institute, Newcastle University, Newcastle upon Tyne, UK

[8]Great North Children's Hospital, Paediatric Intensive Care Unit, Newcastle upon Tyne Hospitals NHS Foundation Trust, Newcastle upon Tyne, United Kingdom.

LMU Munich Partner (Germany)

Principal Investigator:

Ulrich von Both[1,2] MD, FRCPCH (all activities)

Research group:

Laura Kolberg[1] MSc (all activities)

Manuela Zwerenz[1] MSc, Judith Buschbeck[1] PhD

Clinical recruitment partners (in alphabetical order):

Christoph Bidlingmaier[3], Vera Binder[4], Katharina Danhauser[5], Nikolaus Haas[10], Matthias Griese[6], Tobias Feuchtinger[4], Julia Keil[9], Matthias Kappler[6], Eberhard Lurz[7], Georg Muench[8], Karl Reiter[9], Carola Schoen[9]

Author Affiliations:

[1]Div. Paediatric Infectious Diseases, Hauner Children's Hospital, University Hospital, Ludwig Maximilians University (LMU), Munich, Germany

[2]German Center for Infection Research (DZIF), Partner Site Munich, Munich, Germany

[3]Div. of General Paediatrics, [4]Div. Paediatric Haematology & Oncology, [5]Div. of Paediatric Rheumatology, [6]Div. of Paediatric Pulmonology, [7]Div. of Paediatric Gastroenterology, [8]Neonatal Intensive Care Unit, [9]Paediatric Intensive Care Unit Hauner Children's Hospital, University Hospital, Ludwig Maximilians University (LMU), Munich, Germany, [10]Department Pediatric Cardiology and Pediatric Intensive Care, University Hospital, Ludwig Maximilians University (LMU), Munich, Germany

bioMérieux, France

Principal Investigator:
François Mallet[1,2, 3]

Research Group:
Karen Brengel-Pesce[1,2, 3]
Alexandre Pachot[1]
Marine Mommert[1,2]

[1]Open Innovation & Partnerships (OIP), bioMérieux S.A., Marcy l'Etoile, France

[2] Joint research unit Hospice Civils de Lyon - bioMérieux, Centre Hospitalier Lyon Sud, 165 Chemin du Grand Revoyet, 69310 Pierre-Bénite, France

[3] EA 7426 Pathophysiology of Injury-induced Immunosuppression, University of Lyon1-Hospices Civils de Lyon-bioMérieux, Hôpital Edouard Herriot, 5 Place d'Arsonval, 69437 Lyon Cedex 3, France

Department of Infectious Diseases, University Medical Centre Ljubljana, Slovenia

Principal Investigator:

Marko Pokorn[1,2,3] MD, PhD

Research Group:

Mojca Kolnik[1] MD, Katarina Vincek[1] MD, Tina Plankar Srovin[1] MD, PhD, Natalija Bahovec[1] MD, Petra Prunk[1] MD, Veronika Osterman[1] MD, Tanja Avramoska[1] MD

Affiliations:

[1]Department of Infectious Diseases, University Medical Centre Ljubljana, Japljeva 2, SI-1525 Ljubljana, Slovenia

[2]University Childrens' Hospital, University Medical Centre Ljubljana, Ljubljana, Slovenia

[3]Department of Infectious Diseases and Epidemiology, Faculty of Medicine, University of Ljubljana, Slovenia

Amsterdam, Academic Medical Hospital & Sanquin Research Institute (NL)

Principal Investigator:

Taco Kuijpers [1,2]

Co-investigators

Ilse Jongerius [2]

Recruitment team (EUCLIDS, PERFORM):

J.M. van den Berg[1], D. Schonenberg[1], A.M. Barendregt[1], D. Pajkrt[1], M. van der Kuip[1,3], A.M. van Furth[1,3]

Students PERFORM

Evelien Sprenkeler [2], Judith Zandstra [2],

Technical support PERFORM

G. van Mierlo [2], J. Geissler [2]

Author Affiliations:

[1] Amsterdam University Medical Center (Amsterdam UMC), location Academic Medical Center (AMC), Dept of Pediatric Immunology, Rheumatology and Infectious Diseases, University of Amsterdam, Amsterdam, the Netherlands

[2] Sanquin Research Institute, & Landsteiner Laboratory at the AMC, University of Amsterdam, Amsterdam, the Netherlands.

[3] Amsterdam University Medical Center (Amsterdam UMC), location Vrije Universiteit Medical Center (VUMC), Dept of Pediatric Infectious Diseases and Immunology, Free University (VU), Amsterdam, the Netherlands (former affiliation)
